# Five-minute Apgar score ≤ 5 and Molar Incisor Hypomineralisation (MIH) – a case control study

**DOI:** 10.1186/s12903-016-0253-5

**Published:** 2016-07-22

**Authors:** Rivan Sidaly, Andreas Schmalfuss, Anne B. Skaare, Amer Sehic, Tom Stiris, Ivar Espelid

**Affiliations:** Department of Biomaterials, Institute of Clinical Dentistry, Faculty of Dentistry, University of Oslo, Oslo, Norway; Department of Clinical Dentistry, UiT The Arctic University of Norway, Tromsø, Norway; Department of Paediatric Dentistry and Behavioural Science, Institute of Clinical Dentistry, Faculty of Dentistry, University of Oslo, Oslo, Norway; Institute of Oral Biology, Faculty of Dentistry, University of Oslo, Oslo, Norway; Department of Neonatology, Oslo University Hospital, Oslo, Norway; Department of Biomaterials, Faculty of Dentistry, PO Box 1109, Blindern, N-0317 Oslo Norway

**Keywords:** Ameloblasts, Asphyxia, Apgar score, Enamel, Molar incisor hypomineralisation

## Abstract

**Background:**

The aetiology of molar incisor hypomineralisation (MIH) is unclear. The asymmetric distribution of MIH in the dentition may indicate that an insult of short duration that affects ameloblasts at a vulnerable stage could be a causative factor. Apgar ≤ 5 at 5 min may indicate asphyxia (hypoxic-ischemic insult) during birth. It was hypnotised that low Apgar score during birth may cause MIH. The present study aimed to examine a possible association between Apgar ≤ 5 at 5 min and the occurrence of MIH.

**Method:**

Two study groups were selected for examination. The cases comprised 67 children aged 8–10 years born with Apgar score equal to or below 5 after 5 min. The control group comprised 157 age-matched healthy children. First permanent molars, second primary molars and all permanent incisors were examined in all children. Clinical examination was undertaken by two calibrated examiners and intraoral close-up photographs of the teeth were later evaluated by three calibrated and blinded clinicians. Demarcated opacities, post-eruptive breakdown, atypical restorations and extractions due to MIH, according to the criteria of the European Association of Paediatric Dentistry, were assessed.

**Results:**

The prevalence of MIH did not differ between the two groups. A chi-square test failed to confirm any statistically significant relationship between 5-min Apgar scores and MIH occurrence. In addition, there was no statistically significant relationship between the number of affected first permanent molars in cases and controls.

**Conclusion:**

There was no association between Apgar ≤ 5 at 5 min and the occurrence of MIH.

## Background

The regenerative capability of dental enamel is fundamentally limited due to apoptosis of ameloblasts following maturation of the tissue. Disturbances in the function of ameloblasts during tooth development may therefore result in permanent defects. As development of the first primary tooth begins in the fourth week *in utero* and the root development of the wisdom teeth is completed around the age of 20 years, teeth serve a role similar to a “flight recorder” that covers a long time period. From this “record,” the clinician can judge roughly when the disturbance occurred, and the appearance of the defects may in some cases give clues as to the aetiological factor. Thus, the tooth enamel often acts as a repository of information on systemic insults received during development [1].

Molar incisor hypomineralisation (MIH), as defined by Weerheijm et al. [2] in the early 2000s, describes a developmentally derived enamel hypomineralisation affecting 1 to 4 first permanent molars (FPMs) and frequently also permanent incisors. Clinically, the enamel defects of MIH present demarcated opacities that vary in colour from white to yellow/brown with a more or less sharp demarcation between the affected and sound enamel [3]. The asymmetrical occurrence of MIH molars within individuals suggests that the insult causing defective enamel is of short duration and affects ameloblasts at a critical phase [4]. Any temporary or permanent interruption of the ameloblast function depending on the time of insult, may cause enamel hypoplasia or hypomineralisation [4, 5]. Defects occurring in infancy can be diagnosed many years later when the teeth have erupted, as the enamel does not undergo remodeling. The aetiology is complex and there is insufficient evidence in the literature regarding possible factors that may cause these demarcated enamel defects. Some of these are environmental factors with systemic effects. These may include prenatal, perinatal and childhood medical conditions, but an underlying genetic predisposition cannot be excluded [6–8].

An old study of 102 children with neonatal asphyxia showed that 27 % had enamel defects compared with a control group (*n* = 56) with corresponding prevalence of 13 %. A possible relationship between neonatal asphyxia and enamel disturbances could not be ruled out, but the difference was not statistically significant [9]. A questionnaire study among parents of 10-year-old children in Iran revealed that 15 % (*n* = 144) of the children had serious illness during the first month after birth. In this group, the Apgar score < 7 was statistically significantly related with occurrence of developmental defects in permanent teeth (OR 2.32) [10]. The Apgar scoring system is used to assess the newborn immediately after delivery [11]. Low Apgar score may be indication of birth asphyxia. It has been suggested that the length of time it takes to reach Apgar score of 7 is a rough indication of severity of asphyxia [12]. Also low Apgar score at 5 min has been demonstrated to have some predictive value on neurodevelopmental outcome [13, 14] and a recent publication [15] showed that low Apgar score at 5 min was strongly associated with the risk of neonatal and infant death.

Maternal hypoxia during the latter stage of pregnancy has been demonstrated to disturb amelogenesis in the rat foetus [16]. Epidemiological studies indicate that the function of ameloblasts might be affected during human preterm birth [17, 18]. It cannot be excluded that low oxygen level plays a role in such cases. During asphyxia, the combination of the decrease in oxygen supply (hypoxia) and blood supply (ischemia) results in a cascade of biochemical changes that may lead to neuronal cell death and brain damage [19].

Animal studies have shown that hypoxic insult may result in both quantitative and qualitative defects in the enamel, reflecting the vulnerability of ameloblasts toward severe hypoxia. Rats subjected to hypoxia exhibited enamel aberrations in the form of hypoplasia [20], whereas both hypomineralisation and hypoplasia were observed in mice following acute hypoxic insult [21]. The asymmetric distribution of MIH in the dentition may indicate that an insult of short duration which affects ameloblasts in a vulnerable stage could be a causative factor.

The objective of this study was to test the null hypothesis that Apgar ≤ 5 does not increase the risk to develop MIH in humans.

## Methods

The study protocol was approved by the Regional Committee for Medical and Health Research (REK) and informed consent was signed by each child’s parents prior to data collection.

### Sample selection

**Fig. 1:**
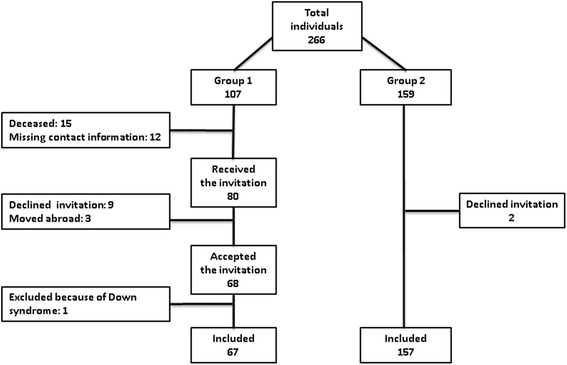
Flow chart of individuals enrolled in the study

This was a cross sectional, case-control study, based on clinical and photographic examinations of 8–10 year old children (*n* = 266) (Fig. 1). Two groups of children were investigated, one group diagnosed with birth asphyxia defined as Apgar score ≤ 5 (Group 1) 5 min after delivery and one healthy control group (Group 2).

Group 1 (cases) comprised all children born 2004-6 admitted to the Neonatal Intensive Care, Ullevål, Oslo University Hospital, with an Apgar score ≤ 5 at 5 min. Originally, this group comprised 107 children of whom 80 were invited to participate (Fig. 1). The national population register (Folkeregisteret) was contacted to confirm the contact details obtained from the medical records. Group 2 (controls) comprised of children healthy at birth attending the Public Dental Service (PDS) in Lillestrøm in the county of Akershus. The controls were age-matched with the cases (born 2004-6). The exclusion criteria were children with genetic syndromes and malformations diagnosed in the neonatal period.

### Data collection and analysis

**Fig. 2:**
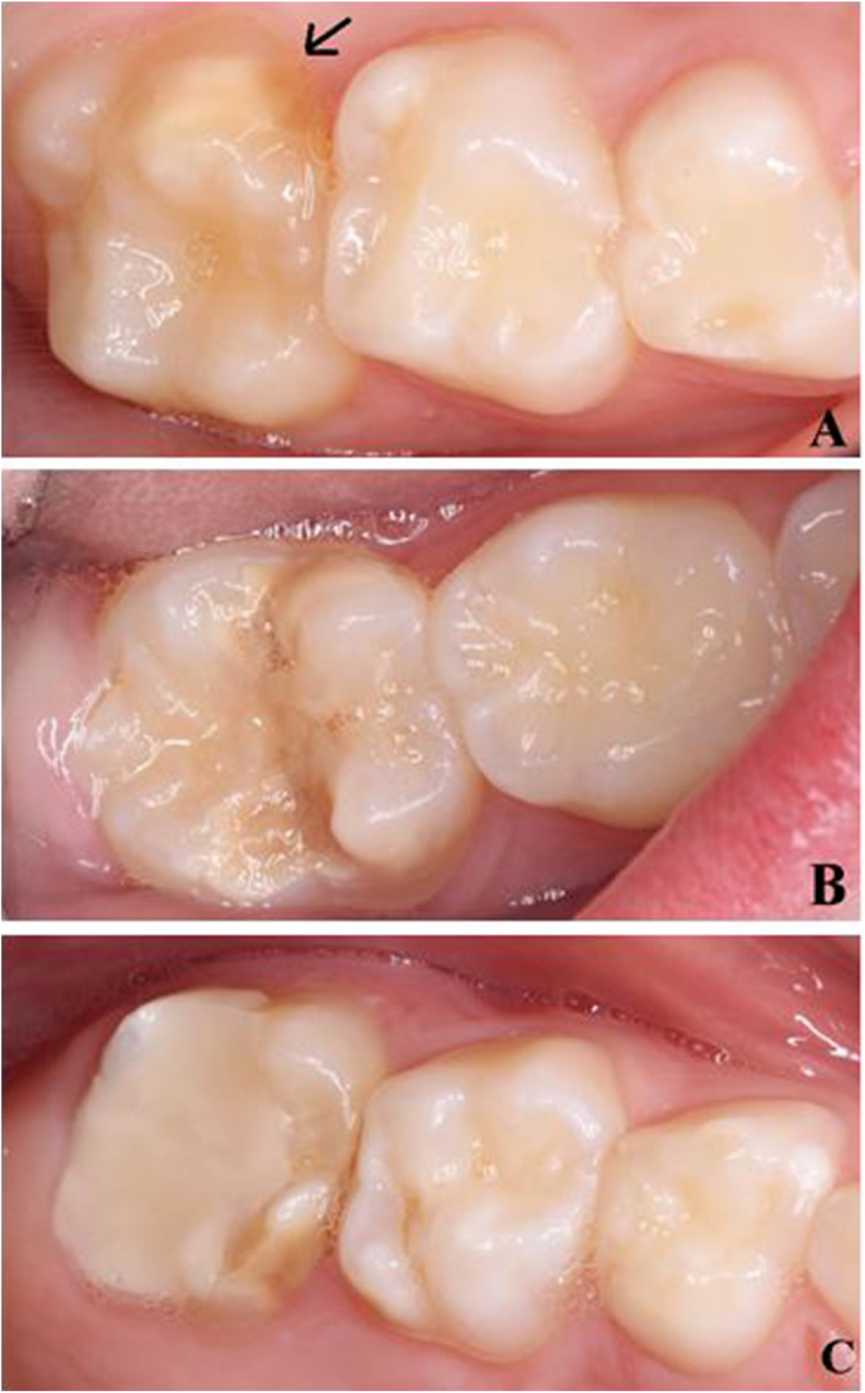
Examples of affected first permanent molar from three individuals with MIH enrolled in the study. Defects varies in severity from diffuse yellow opacities (*black arrow*) in tooth 16 (**a**) to more severe changes due to posteruptive breakage (PEB) in tooth 46 (**b**) and atypical filling due to severe MIH in tooth 26 (**c**)

A structured medical history questionnaire was sent or given to the parents of the participating children. The questionnaire included data on maternal health, pregnancy, delivery, prematurity, breastfeeding, use of antibiotics and infection during early childhood, as described in previous studies [22, 23]. The children were examined in the dental clinic by two dentists (AS and RS) for the presence and severity of MIH. Dental lighting, mirrors and blunt probes were used. When there was disagreement consensus was reached through discussion. Prior to the examination, a calibration exercise was conducted among all the examiners (RS, AS, ABS and IE) using clinical photographs of patients selected from the patient records in the Department of Paediatric Dentistry, University of Oslo. The examiners applied the diagnostic criteria that were agreed on at a workshop organised by the European Academy of Paediatric Dentistry [24]. All second primary molars and index teeth which include first permanent molars and all permanent incisors were examined for demarcated opacity, post eruptive enamel breakdown (PEB), atypical restorations replacing affected enamel and extractions due to MIH (Fig. 2). Opacities < 1 mm in diameter were not recorded. The term Molar Incisor Hypomineralisation was used for dentitions with one or more hypomineralised FPMs with or without hypomineralised permanent incisors [25]. When more than one defect occurred on a tooth surface, the most severe was recorded. Sum scores of recordings on FPMs were calculated to express the severity of MIH in each individual. For each person with MIH, the sum score theoretically could range from 1 to 8, as each tooth received score 1 for opacity or 2 for PEB, atypical filling or extraction due to MIH. The sum score is the total of 4 single scores for each FPM. The final diagnosis of MIH was based on consensus among the observers. In some cases, the quality of the photographs was not optimal due to poor patient compliance and the clinical diagnosis was taken into account.

After the clinical examination, a digital camera (Canon EOS 60D, Canon INC., Japan) fitted with a macro-lens (Canon Macro Lens EF 100 mm 1:2,8 USM, Japan) and a ring flash (Canon Ring Lite MR -14EX, Japan) was used to capture macro-photographs of the dentition. Most of the photographs were taken by one photographer according to a standardised protocol. Seven photographs were taken in the following order: the buccal surfaces of the teeth in the first and fourth quadrant (#1), the corresponding surfaces in the second and third quadrant (#2), the buccal surfaces of the upper and lower anterior teeth (#3), and the occlusal surfaces of the upper teeth (#4 & 5) and lower teeth (#6 & 7). All the photographs were immediately examined for photographic quality, and if necessary photographs were repeated. The photographs were displayed on Sony 55” TV-monitor (Sony Model no.KDL 55WB02A LCD-TV, Sony Corporation, Japan) in a room with masked windows and dimmed room lighting. The intraoral photographs were then evaluated for enamel defects by three independent and blinded observers (AS, ABS and IE). One month later, the clinical photographs of a subsample of children (*n* = 35, 15.5 %) were re-examined in the same room under identical lighting conditions on the same TV monitor. Cohen’s kappa was calculated for inter- and intra-examiner agreement.

### Data handling

Data from questionnaires were tabulated and analysed using the Statistical Package for Social Sciences 22.0 for Windows (SPSS Inc., Chicago, Illinois, USA). Chi-square test was used for comparison. Significance was set at *p* < 0.05.

## Results

Of 107 children in group 1, the parents of 54 children did not receive an invitation (Fig. 1). Eighty children/parents were eligible and therefore invited to take part in the study. Sixty-eight parents accepted the invitation (85.0 %). Out of the 159 controls invited to participate, two parents (1.3 %) declined (Fig. 1). The distribution of boys and girls in group 1 was 53.0 and 47.0 %, respectively and the corresponding rates in the control group were 49.7 and 50.3 %. The mean age in group 1 was 9.0 years (SD 0.84, range 8–10), and 9.0 years (SD 0.80, range 8–10) in group 2.

Based on the clinical examination, the prevalence of MIH in group 1 was 23.5 and 25.4 % in group 2 (*p* = 0.76). Some examples of MIH teeth found among the included individuals are shown in Fig. 2. The results of the joint decisions, based on examination of the intraoral photographs, showed that the MIH prevalence in the asphyxia group was 29.4 % and in the control group 31.2 % (*p* = 0.79). Furthermore, there was a higher proportion of controls (5.7 %) than cases (2.9 %) who had a MIH sum score between 5 and 8, but there was no statistically significant differences between the groups (Table 1) or in the average severity score per tooth between the two groups (*p* = 0.42). There were no statistically significant differences in the distribution of affected index teeth and second primary molars in individuals from both groups (Table 2). Analysis of medical records of the children in group 1 who did not wish to participate showed that there was no increased morbidity during the first year of life.

**Table 1:** Distribution of individuals according to MIH score in the Group 1 (Apgar score ≤ 5) and Group 2 (controls)

	Group		
MIH score	Group 1	Group 2	*p*-*value*
	(*n* = 68)	(*n* = 157)	
0	69.1	67.5	0.94
1–2	16.2	14.4	0.69
3–4	11.8	12.4	0.05
5–8	2.9	5.7	0.40
Total	100.0	100.0	

MIH score is cumulative score for all four FPMs in each individual. The scores for each tooth may vary from 0 (no MIH) to 2 according to severity. No statistically significant differences between groups

**Table 2:** Distribution of affected index teeth and second primary molars in individuals with Apgar score ≤ 5 (Group 1) compared to control individuals (Group 2)

	Group		
Affected tooth group	Group 1	Group 2	*p*-*value*
Upper FPM	22.8	21.3	0.63
Lower FPM	11.0	18.5	0.13
Upper central incisors	5.3	6.1	0.61
Upper lateral incisors	3.5	3.8	0.54
Upper second primary molars	8.2	5.4	0.43
Lower central incisors	2.9	2.5	0.64
Lower lateral incisors	3.0	2.6	0.77
Lower second primary molars	8.2	4.1	0.08

No statistically significant differences between groups

There was no statistically significant difference in MIH prevalence between girls and boys neither in group 1 (*p* = 0.75) nor group 2 (*p* = 0.57). Inter-examiner and intra-examiner agreement for MIH diagnosis at the patient level, measured by Cohen’s kappa, was in the range 0.60–0.78 (mean 0.69) and 0.55–0.76 (mean 0.65), respectively.

Analyses of the self-reported health information (questionnaires) revealed that there was no statistically significant association with MIH and health related variables between groups.

## Discussion

The null hypothesis to be tested was that Apgar ≤ 5 at 5 min, does not increase the risk of developing MIH. The hypothesis was accepted as there was no statistically significant difference in the prevalence of MIH between the study group and controls based on clinical and photographic examinations. Further, the number of affected FPMs or the severity of MIH on affected teeth did not differ between groups and these findings support the rejection of the null hypothesis.

Many prevalence studies have been carried out in different countries using the new definition of diagnostic criteria for MIH [3] and this definition was also used in the present study. Large variations in prevalence have been reported, ranging from 2.4–40.2 % [26]. This wide range may be explained by differences in recording methods, different ages or different study populations [27, 28]. In some countries, generally high caries experience may mask the true prevalence of MIH [28]. The clinical examination showed a lower prevalence compared with recordings based on photographs. The prevalence of MIH in the present control group was found 25 %, based on the clinical examination, corresponds well with findings from Finland (25 %), Denmark (15–25 %) (Esmark 1995 cited by Weerheijm 2003 [25]) and Sweden (18 %) [29, 30]. However, these studies were published before the criteria by European Academy of Pediatric Dentistry were established [24] and some deviations according to variability in criteria applied might be expected. Digital photography with magnification on the monitor allows detailed examination of enamel [31], possibly explaining the higher prevalence reported with this method in the present study. In addition, the use of photographs facilitates randomisation and blinding, limiting observer bias, which is important in a study like this. As in other studies, there was no statistically significant difference in prevalence between the sexes in either group [32–34]. Furthermore, the relative distribution between tooth groups and upper and lower jaw are consistent with the findings that have been reported by Lygidakis et al. [35].

Decreases in oxygen supply are known to initiate adaptive mechanisms designed to maintain cellular activity at a minimum level. The failure of these mechanisms during hypoxia results in cellular dysfunction and can lead to irreversible cell damage [36]. In general, cellular adaptations to hypoxia rely on the transcription factor hypoxia-inducible factor (HIF), which is inactive when oxygen is abundant but is activated under hypoxic conditions [37, 38]. This may also be likely for the affected ameloblasts exposed to hypoxia. Further, it may be speculated that compensatory mechanisms like increased expression of HIF-1α, which is shown to be up-regulated in ameloblast cells following hypoxia [39], may be the reason for not developing MIH in patients with mild hypoxia.

It is known that combined Apgar score and increased acidosis is more indicative of the severity of asphyxia. In 2004-2006, umbilical blood gases were not analysed routinely and in the present study, an Apgar score ≤ 5 at 5 min was used as entry criterion since it was used for hospital admission at the time. Thus the hypoxia may not have been severe enough to induce changes in the ameloblasts to cause MIH.

Grahnén et al. (1969), in their study, were unable to demonstrate a significant difference in enamel hypoplasia of the primary teeth [9], but this study had some weaknesses: the cases included low birth weight children, while the controls did not. Furthermore, the criteria for diagnosis of asphyxia were not stringent.

Attrition of participants in group 1 could be due to unavailability, inaccessibility or dental anxiety. Some patients did not survive, which results in lack of information about enamel disturbances in this group. It may therefore be speculated that the results represent an underestimation of MIH. It is also tempting to suggest that the hypoxia was a momentary insult that possibly affected the ameloblasts in a very short but vulnerable stage, which clinically appears in the form of asymmetrical defects influencing the FPMs [40]. This is in accordance with our recent findings demonstrating that there is a considerable variation in ameloblast reaction to hypoxia [21].

Several studies have suggested that hypoxia may affect ameloblast function adversely, both in the rat [16, 41], the hamster [42] and human [17, 43–46]. The results from the present clinical investigation were not consistent with our findings recently reported in an animal study [21]. We have previously demonstrated that a short episode of induced severe hypoxia in adult mice left its mark on mouse incisors in the form of enamel defects. The position and character of the defect were related to the functional stage of the affected ameloblasts. All the cells of the enamel organ, i.e. preameloblasts, secretory ameloblasts, transitional ameloblasts, maturation ameloblasts and pigmentation ameloblasts were subjected to the hypoxia, but seemed to respond differently. Affected enamel showed hypoplasia with or without hypomineralisation [21].

## Conclusions

Based on the results of the present cross-sectional, case-control study, it can be concluded that a low Apgar score at 5 min was not associated with the appearance of MIH or the number of affected FPMs. Bearing in mind that this was probably a mild insult, further investigation of individuals with lower Apgar score would shed more light on the role of hypoxia in the etiology of MIH. In future studies low Apgar score should be associated with other clinical markers of asphyxia like increased acidosis.

## Abbreviations

FPMs, first permanent molars; HIF, hypoxia-inducible factor; MIH, molar incisor hypomineralisation; PEB, post eruptive enamel breakdown; PDS, Public Dental Service; REK, Regional Committee for Medical and Health Research
